# Anisotropic Psychophysical Trends in the Discrimination of Tactile Direction in a Precision Grip

**DOI:** 10.3389/fnins.2020.576020

**Published:** 2021-01-12

**Authors:** Justin Tanner, Naomi Newman, Stephen Helms Tillery

**Affiliations:** ^1^School of Biological and Health Systems Engineering, Arizona State University, Tempe, AZ, United States; ^2^University Medical Center, Banner Health, Phoenix, AZ, United States

**Keywords:** precision grip, psychophysics, sensitivity, bias, anisotropic

## Abstract

Tactile cues arising from interactions with objects have a sense of directionality which affects grasp. Low latency responses to varied grip perturbations indicate that grasp safety margins are exaggerated in certain directions and conditions. In a grip with the ulnar-radial axis vertical, evidence suggests that distal and downward directions are more sensitive to task parameters and have larger safety margins. This suggests that, for the purpose of applying forces with the fingers, reference frames with respect to the hand and gravity are both in operation. In this experiment, we examined human sensitivities to the direction of tactile movement in the context of precision grip in orientations either orthogonal to or parallel to gravity. Subjects performed a two-alternative-forced-choice task involving a textured cube which moved orthogonal to their grip axis. Subjects’ arms were placed in a brace that allowed for finger movement but minimized arm movement. Movement of thumb and index joints were monitored via PhaseSpace motion capture. The subject was presented with a textured cube and instructed to lightly grasp the cube, as if it were slipping. In each trial the object was first translated 1 cm in 0° (proximal), 90° (radial), 180° (distal), or 270° (ulnar) and returned to its origin. This primary stimulus was immediately followed by a 10 mm secondary stimulus at a random 5° interval between −30° and 30° of the primary stimulus. Response from the subject after each pair of stimuli indicated whether the test direction felt the same as or different from the primary stimulus. Traditional bias and sensitivity analyses did not provide conclusive results but suggested that performance is best in the ulnar-radial axis regardless of gravity. Modeling of the response curve generated a detection threshold for each primary stimulus. Lower thresholds, indicating improved detection, persisted in the ulnar-radial axis. Anisotropic thresholds of increased detection appear to coincide with digit displacement and appear to be independent of the grasp orientation.

## Introduction

Achieving success in motor tasks requires viable and interpretable somatosensation, especially as the task’s nature becomes finer. Removing somatosensation severely hinders motor ability, leaving a person to rely on visual feedback or learned motor patterns, both incurring high levels of error (Marsden et al., 1984). Even with complete somatosensory functionality, there are limits in perceptual abilities associated with fine motor tasks as they provide incomplete data that must be subjectively interpreted. Understanding these subjective limits will help identify the properties of normal somatosensation. A key aspect of successfully performing accurate grip movements is discriminating finite differences between movements across fingertips. The directional element of tactile input is useful in informing grasp intent and response to perturbations. This directional tactile discrimination plays an important role in catching falling objects and adjusting grip on moving objects.

Studies of angular discrimination have concentrated on passive poses, such as a hand or finger placed facing down (Webster et al., 2005). These investigated the absolute threshold of directional discrimination in the coronal plane utilizing a passive touch in which subjects placed their index fingers on a rotating ball device. This device’s direction varied in 5° increments and subjects identified the direction as either “angled” or “straight.” The average least noticeable angular difference in slip direction was determined to be between 20° and 25°. A similar study incorporating various textures found this least noticeable angular difference to be between 3.6° and 11.7°, depending on the surface texture (Salada et al., 2004). While the information provided by texture from movement across the relaxed hand is useful in the exploration and identification of new objects, directional discrimination is more intrinsically related to active tasks. In addition, it has been shown that proprioception from large arm movements affects the ability to determine slip speed (Salada et al., 2004), so it is important to limit the inclusion of proprioceptive information as much as possible by limiting movement proximal to the wrist when investigating perceptual thresholds at the fingertips. These referenced studies did not utilize practical hand postures such as precision grips so knowledge regarding this tactile direction discrimination threshold in precision grip is limited. This information is necessary to understand directional discrimination in the context of fine motor actions during practical tasks.

Anisotropic sensitivities of directional discrimination have been observed in numerous studies, but not in precision grip. Psychophysical static groove orientation detection favors grooves oriented perpendicularly to the fingerpad (Essock et al., 1997). Detection and discrimination of grooved indentations scanned across the finger produces increased psychophysical and median nerve responses in the distal-proximal axis (Wheat and Goodwin, 2000). In an investigation of static groove detection anisotropies at different sites, it was found that the fingertip was more sensitive to grooves oriented along the distal-proximal axis, and the finger base was more sensitive along the lateral axis, and the fingerpad was anisotropic (Gibson and Craig, 2005). Finally, primary somatosensory cortex neural activity during discrimination of static indented bars demonstrates tuning in the distal-proximal directions during scanning studies (Bensmaia et al., 2008). These textural features pass across the fingers’ dermal ridges and generate varied vibrational power, activating mechanoreceptors used in such detection (Maeno et al., 1998). At the tip of the finger, neural encoding of force loading direction is also sensitive to the distal direction, noted to be perpendicular to the papillary ridges (Birznieks et al., 2001). With respect to angular slip direction, slip speed, and slip texture, anisotropic sensitivities in detection thresholds favor the distal-proximal direction as opposed to ulnar-radial. However, the studies discriminating scan direction were examined under passive, non-grip tasks which leaves us with an opportunity to explore these trends and properties in an active task.

In an active precision grip, more variables are at work than in passive states, and this creates heightened direction sensitivities which allow for quicker and stronger responses. We can glean some anisotropic trends in this grip setting. Literature reports that a reactionary pinch force to a precision grip stimulus is increased for distally traveling stimuli (Jones and Hunter, 1992). There is also lower grip force latency and greater grip force safety margin in distal directions and in the direction of downward gravity, confirmed by utilizing inverted grip but not an orthogonal grip (Häger-Ross et al., 1996). While these are quantitative measures of our intrinsic grip reactions, they also imply that subjective directional grip discrimination may be biased in certain critically dangerous directions. If these subjective anisotropies are consistent with environmental factors such as gravity, we can infer that our tactile discrimination is externally referenced. However, reducing gravity does not affect grip performance or cyclic loading, but does affect force scaling necessary for appropriate safety margins, suggesting internal reference frames for subjective responses (Augurelle et al., 2003).

The necessary grip force during normal gravity would, however, apply higher shear forces on the finger pad in the direction of gravity and would likely induce lateral finger movement more easily. Since the glabrous skin of the finger pad is anisotropic, with stiffness relating to the orientation of the papillary ridges, movements across these ridges would induce more deformation (Wang and Hayward, 2007). The orientation of the papillary ridges is not consistent across the finger pad, but the center has ridges primarily orthogonal to the ulnar-radial axis. Mechanoreceptor sensitivity seems to follow similar patterns of this anisotropy, showing ridge-orthogonal tuning for SA systems and ridge-parallel tuning for certain mechanoreceptors (Birznieks et al., 2001). Skin stretch is tied to directional detection (Seizova-Cajic et al., 2014), so it is our hypothesis that the axis with more deformation, the radial-ulnar axis, will likely align with the axis of sensitivity.

Directional tactile sensitivities exist in different directions for multiple contexts but can be generally reduced to variable and contextual biomechanical loading. Precision grip tends to not rely on scanning across the finger, so the deformation due to shear forces is likely the method of activation. Axes sensitive to tactile direction in precision grip are unclear, but likely will align with the ulnar-radial axis as it is less stiff and more deformable. Whether those sensitivities are referenced to internal biomechanics or to external effects such as gravity must be jointly determined. An internal reference would support that environmental factors like gravity are superseded by mechanoreceptor information for contextually informing perception, grip structuring, and future planning.

Here we examine the effect of changing the grip orientation with respect to gravity on the perception of slip direction during active grip. We report that the sensitivity to movement direction is largely aligned along the radial-ulnar axis.

## Materials and Methods

Using a precision grip, 14 subjects (8 female, 6 male, 20–32 years old) held a 50 mm cube textured with 60 grit sandpaper that was attached to a six degree of freedom DENSO (Long Beach, CA, United States) VS-G series robotic arm. Each subject was instructed to lightly hold onto the cube, maintaining contact, but not attempting to immobilize the object. Stimuli were delivered to the subject via a custom LabVIEW (National Instruments, Austin, TX, United States)/Python program. Experimental protocols were reviewed and approved by the Institutional Review Board at Arizona State University.

**FIGURE 1:**
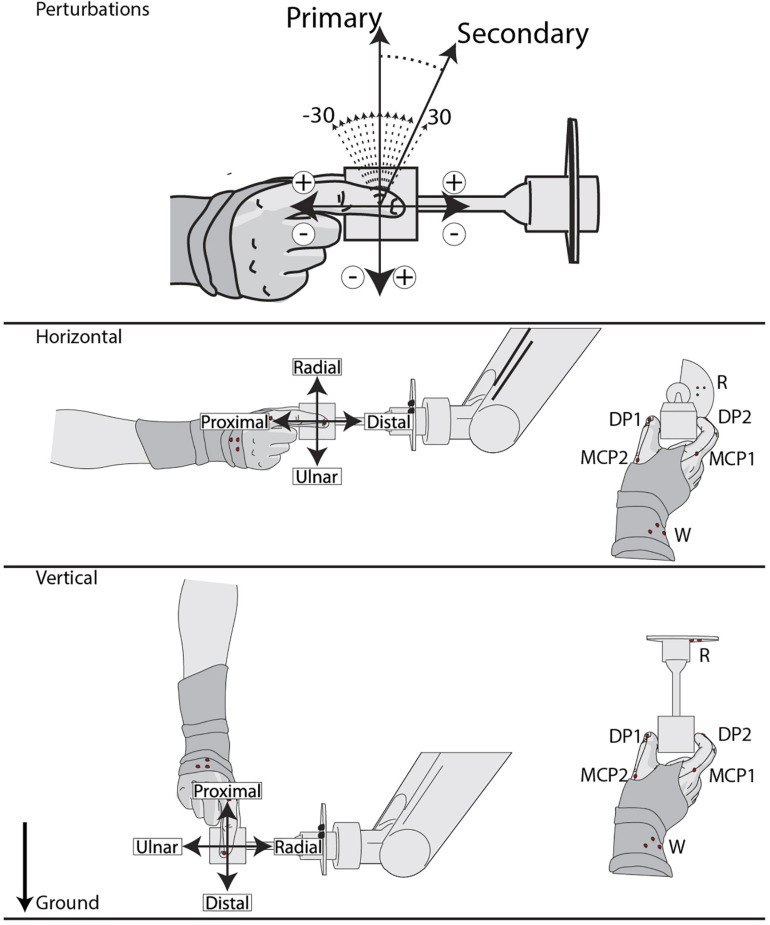
Experimental setup and task. Task: a two-alternative, forced-choice paradigm consisting of both a primary and secondary stimulus, with a randomized 300–700 ms interstimulus interval. The primary stimulus is a 10 mm (20 mm/s) center-out-center movement in the proximal, radial, distal, or ulnar direction. The equidistant and equal velocity secondary stimulus differs within ±30° on 5° increments from the primary stimuli, indicated by respective sign conventions. Each subject responds “Same” or “Different” to the stimuli pair. Grip Orientations: primary stimulus definitions are defined by the rotation of the grip with respect to the ground. PhaseSpace markers on the M, DPs, Wrist, and Robot are also represented as red dots.

Based on a two-alternative, forced-choice task, subjects were presented with two stimuli and asked to determine if they were the “same” or “different.” Each stimulus was a 10 mm movement of the gripped cube at 20 mm/s in varied sagittal directions. A randomized primary stimulus in the proximal, radial, distal, or ulnar direction was followed by a randomized 300–700 ms interstimulus interval. Then a secondary stimulus with a randomized angular difference of ±30° on intervals of 5° was delivered (Figure 1). Subjects were asked to determine whether the stimuli were in the “same” or “different” directions. In order to explore the reference frame of potential grip sensitivities, two grip orientations were used (Figure 1): horizontal (nine subjects; five female, four male) and vertical (five subjects; three female, two male). The primary stimulus definitions were aligned with the arm, and thus rotated by 90° between these two orientations. Grip and task instructions were identical for each grip orientation.

To avoid unwanted visual and proprioceptive feedback, subjects were blindfolded with their wrist mounted in a cushioned brace attached to a rigid frame. Coordinates from a PhaseSpace motion capture unit (PhaseSpace Inc, San Leandro, CA, United States) were referenced to the robotic arm so that the *Y*- and *Z*-axes corresponded with the subject’s sagittal plane. Motion capture markers were placed on the robotic arm, metacarpophalangeal joints (MCP), and the tip of the distal phalanges (DP) of digits 1 and 2 (MCP1, MCP2, DP1, and DP2), and the forearm just proximal to the wrist for each subject (Figure 1). Motion capture marker distance was defined as the maximum sagittal distance for each trial. Movement for each joint was evaluated using a two-way interaction ANOVA with grip orientation and primary stimuli and Tukey-Kramer tests are used for *post hoc* analysis on significant effects.

Sensitivity (d′) and bias (β) definitions were obtained for each primary stimulus using Eqs 1 and 2, respectively (Stanislaw and Todorov, 1999). Equation 1 is also divided into the necessary variables for clarification. Z(H) is the *z*-score conversion of the probability the subject has a hit (H) and identifies a “Different” trial correctly (True Different – TD). Z(F) is the *z*-score conversion of false alarms (F): when the subject identifies any “Same” trial incorrectly (False Same – FS). Sensitivity and bias were evaluated using a two-way interaction ANOVA with grip orientation and primary stimuli.

To determine thresholds of detection, we obtained a Point of Subjective Detection (PSD) by first defining the detection rate (DR) for each secondary stimulus (Eq. 3), with correctly identified trials as true and incorrectly identified trials as false. As shown in Eq. 3, the PSD is the angle where the proportion of true responses exceeds the false responses, i.e., when the DR becomes greater than 50%. The DR values were fit for all subjects grouped and for each subject individually using a second order polynomial regression and solutions for 50% are calculated. These solutions were considered the detection threshold and used to ascribe response trends to specific primary stimuli. Accuracy for each primary stimulus was calculated as the total correct responses of that primary stimulus over its total trials. Increased response accuracy and lower psychophysical PSD provide support for directional anisotropies. Detection thresholds were evaluated using a two-way interaction ANOVA with grip orientation (two levels) and primary stimuli (four levels). In a second analysis, primary stimuli were grouped into primary axes as proximal-distal and radial-ulnar rather than individual directions.

(1) $$d^{\prime }\,\left(P\,r\,i\right)=\frac{Z\,\left(H\right)-Z\,\left(F\right)}{\sqrt{2}},$$

(1a) $$H\,\left(P\,r\,i\right)=\frac{T\,D\,\,\left(P\,r\,i\right)}{T\,D\,\left(P\,r\,i\right)+\,F\,D\,\left(P\,r\,i\right)}$$

(1b) $$F\,\left(P\,r\,i\right)=\frac{F\,S\,\left(P\,r\,i\right)}{T\,S\,\left(P\,r\,i\right)+\,F\,S\,\left(P\,r\,i\right)}$$

(2) $$\beta \,\left(P\,r\,i\right)=\frac{Z\,\left(H\right)+Z\,\left(F\right)}{2}$$

(3) $$D\,R\,\left(P\,r\,i,S\,e\,c\right)=\frac{T\,\left(P\,r\,i,S\,e\,c\right)}{T\,\left(P\,r\,i,S\,e\,c\right)+F\,\left(P\,r\,i,S\,e\,c\right)}$$

## Results

**FIGURE 2:**
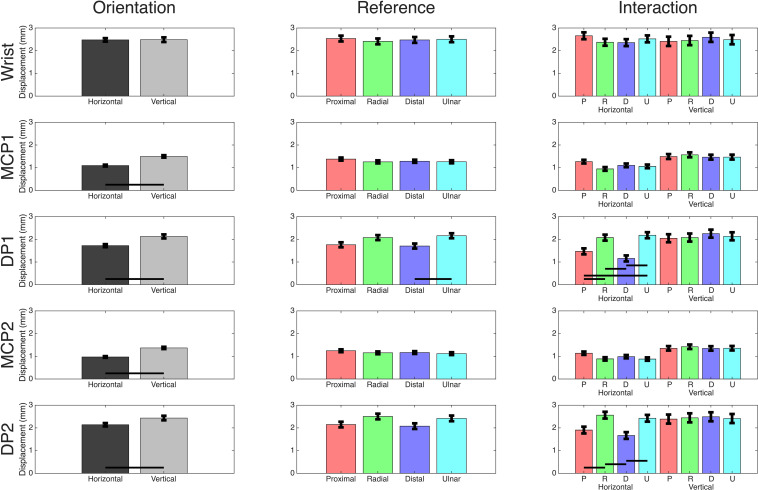
Motion capture comparisons. Mean values for each two-way ANOVA performed on the motion capture marker’s two-way ANOVA using orientation and primary stimuli as effects. Black bars indicate a significant *post hoc* Tukey-Kramer result of *p*<0.05. Comparisons between orientations in the interaction plots are removed as they are not of interest, but significant results between references movements within orientation are shown.

Motion capture data were used to calculate the absolute maximum distance traveled in the sagittal plane for each trial. Significant movement between grip orientation and primary stimulus for each joint of interest was determined by constructing respective two-way ANOVAs (Table 1). The wrist demonstrated no significant displacement for factors nor interactions. In both the thumb (digit 1) and index finger (digit 2), the MCP and tip of the DP had significantly less movement in the horizontal orientation than the vertical. For primary stimuli and the interaction effect, only the DP1 and DP2 produced significant differences. Tukey-Kramer *post hoc* analyses were conducted for these significant effects (Figure 2). Analysis of the primary stimulus factor revealed that the DP1 moved less during distal movements than ulnar movements but did not indicate any significant differences for DP2. As for the interaction effect *post hoc* analyses, DP1 demonstrated significant results in the horizontal orientation, but not the vertical orientation: distal and proximal trials produced less movement than radial and ulnar trials. DP2 was similar, except proximal trials’ movement was not significantly different than ulnar trials. Across all digits and conditions movement was less than 3 mm, less than the 10 mm the gripped cube actually moved. In significant cases, the difference in means ranged from 0.6 to 1.02 mm.

**TABLE 1:** Summary of two-way ANOVA on digit movement.

	Wrist	MCP1	DP1	MCP2	DP2
**Motion capture**					
Orientation	0.947	***p* < 0.005**	**0.0002**	***p* < 0.005**	**0.0178**
		H < V	H < V	H < V	H < V
Primary Movement	0.9131	0.5365	**0.0058**	0.5203	**0.042**
			Significance (Δmm)		No Tukey-Kramer
			D < U (0.453)		
Interaction	0.5963	0.2147	**0.0005**	0.2668	**0.025**
			Significance (Δmm)		Significance (Δmm)
			Horizontal		Horizontal
			P < R (0.6), P < U (0.71)		P < R (0.65)
			D < R (0.91), D < U (1.02)		D < R (0.89), D < U (0.75)

*Individual two-way ANOVAs were constructed for each finger’s MCP and DP markers as well as the wrist. Any significant factor is bolded and accompanied by the significant *post hoc* comparisons and relevant differences in means, except for the DP2 Primary Stimulus effect, which did not provide significant *post hoc* results.*

Bias (β) and sensitivity (d′) values were calculated for each set of grip orientation and primary stimuli trials. A two-way interaction ANOVA was performed with grip orientation and primary stimuli. No significant differences for bias nor sensitivity were found. Distal and proximal axes mostly exhibit increased β values, implying a preference of responding “different” in these directions. A higher bias would suggest a decreased ability to detect “same” trials. In addition, the d′ values are slightly higher for the distal-proximal axis in the horizontal grip and the vertical grip. With no significant β or d′ results, we explored threshold of detection.

**FIGURE 3:**
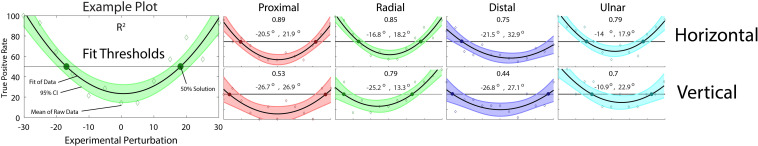
Polynomial regression fits of response sensitivity. For each primary stimulus in each grip condition, detection rate (DR) is calculated as a secondary stimuli’s True Positives over the sum of respective True Positives (TP) and False Negatives (FN). The PSD mark represents where TP ≥ FN. Subjects who showed a DR ≤ 50% for ±30° or 0° secondary stimuli were excluded due to lack of attention or focus during the experiment. For all subjects, the mean DR is overlaid with a second order polynomial regression fit and respective 95% confidence intervals. Solutions for 50%, and *R*^2^ are given for each plot.

The task was designed to exceed existing reports of directional discrimination limits and required the subjects’ attention. Subjects whose DR was less than 50% for ±30° or 0° trials were excluded, and the high error was attributed to lack of attention or task vigilance. These rules revealed two horizontal grip exclusions and one vertical grip exclusion. To determine the PSD, data were modeled with second order polynomial regressions, 95% confidence intervals were calculated, and solutions for 50% DR calculated (Figure 3). Using the Akaike Information Criterion (AIC), it was determined that utilizing a fourth order polynomial model overfits the data and second order models provide a higher quality fit. The full results are summarized in Table 2, including accuracy calculations for each primary stimulus under each grip treatment and AIC values for the second and fourth order models. The *R*^2^ values included are representative of the chosen second order model. Determined PSD values occurred within 10.9° to 32.9°, depending on axis and grip orientation.

**TABLE 2:** Summary of analytical results for each primary stimulus under grip orientations.

		***Primary stimulus***			
		**Proximal**	**Radial**	**Distal**	**Ulnar**
Horizontal grip	+	21.9	18.2	32.9	17.9
*PSD*	−	20.5	16.8	21.5	14
	Δ	42.4	35	54.4	31.9
	AIC (2°)	57	58.5	52.7	57
	AIC (4°)	61.1	62.9	56.7	61.6
	*R*^2^	0.89	0.85	0.75	0.79
	Accuracy	47.8	59.3	37.3	63.1
	Standard error (*n* = 7)	1.73	1.04	2.45	1.34
Vertical grip	+	26.9	13.3	27.1	22.9
*PSD*	−	26.7	25.2	26.8	10.9
	Δ	53.6	38.5	53.9	33.8
	AIC (2°)	35.4	38.5	34	37.4
	AIC (4°)	39.5	42.8	38.9	41.6
	*R*^2^	0.53	0.79	0.44	0.7
	Accuracy	37.5	54.8	36.5	59.6
	Standard error (*n* = 4)	4.1	0.9	3.18	4.26

*Accuracy: correct responses over total trials for each primary stimulus. Detection thresholds: (+) and (−) indicate the solutions at the PSD for the polynomial fit of the mean subject response, respective to the sign convention indicated in Figure 1. Δ is the range between PSDs. Akaike Information Criterion (AIC) values are listed for second and fourth order polynomials. In all cases, the second order models had lower AIC values and therefore are considered better fits. *R*^2^: fit coefficient of determination for the second order polynomial.*

**FIGURE 4:**
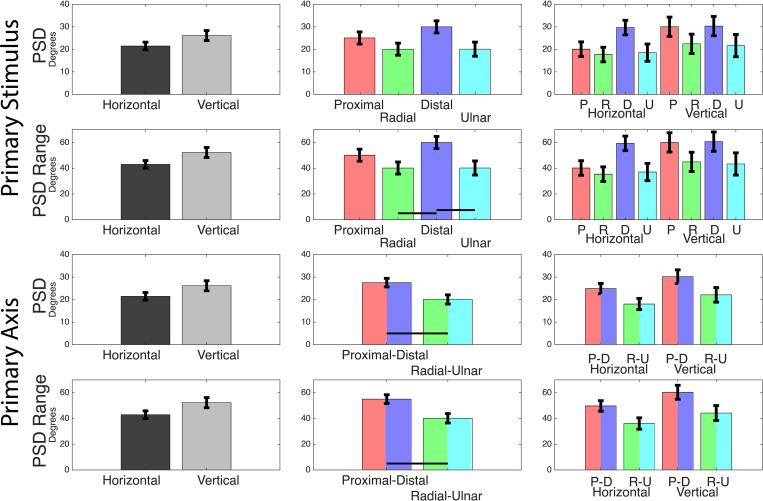
Points of subjective detection. Mean values for each two-way ANOVA performed on the calculated PSD values and PSD windows. Tests were performed with primary stimuli grouped into four levels (movements) and two levels (axes). The latter grouped radial with ulnar and grouped distal with proximal. Black bars indicate a significant *post hoc* Tukey-Kramer result of *p* < 0.05.

Using second order models to fit individual subject’s DR values, we calculated PSD values for each primary stimulus. A two-way ANOVA of PSD values using grip orientation and primary stimuli (four levels) reports that primary stimuli is a significant effect. *Post hoc* Tukey-Kramer analysis reports that distal thresholds are greater than radial and ulnar. A second two-way ANOVA of grip orientation and primary axis rather than stimuli (two levels) reports that primary axis is a significant effect. *Post hoc* Tukey-Kramer analysis reports that distal-proximal thresholds less than radial-ulnar thresholds (Figure 4 and Table 3). The PSD values in Table 2 also imply some asymmetry along certain axes, primarily the radial-ulnar axis, where the positive and negative PSD values deviate in magnitude. Due to this axial asymmetry, it is hard to define specific PSDs for the directional discrimination, but the range within the determined PSDs informs us of windows that would provide subjective uncertainty. A two-way ANOVA of the window ranges using grip orientation and primary stimuli reports that primary stimuli is a significant effect. *Post hoc* Tukey-Kramer analysis reports that the distal uncertainty windows are greater than radial and ulnar. A second two-way ANOVA of the window ranges using grip orientation and primary axis reports that primary axis is a significant effect. *Post hoc* Tukey-Kramer analysis reports that distal-proximal thresholds greater than radial-ulnar thresholds (Figure 4 and Table 3).

**TABLE 3:** Summary of two-way ANOVA on points of subjective detection.

**Detection thresholds**	**Primary direction**		**Primary axis**	
	**PSD**	**PSD range**	**PSD**	**PSD range**
Orientation	0.1029	0.0679	0.0969	0.0678
Primary movement	**0.0385**	**0.0183**	**0.0093**	**0.005**
	No Tukey-Kramer	Significance (Δ°)	Significance (Δ°)	Significance (Δ°)
		D > R (19.84), D > U (19.82)	P-D > R-U (7.45)	P-D > R-U (14.90)
Interaction	0.6654	0.5659	0.8254	0.8057

*Two-way ANOVAs were constructed for the calculated PSD values and PSD window ranges. This is performed grouping trials into primary directions (four levels) and primary axes. Any significant factor is bolded and accompanied by the significant *post hoc* comparisons and relevant differences in means, except for the primary direction ANOVA for the PSD values, which did not provide significant *post hoc* results.*

## Discussion

The β and d′ calculations offered valuable suggestions in directional bias and sensitivity. Specifically, that there is a subjective bias to answer “different” and an increased sensitivity in the distal-proximal axis, largely regardless of grip orientation. These suggestions were statistically insignificant and further analysis contradicted the suggested trend, as Points of Subjective Detection (PSD) indicated a significant anisotropy where discrimination improved in the radial-ulnar axis. Distal-proximal PSD values were near literature values, between 20.5 and 32.9°, but the radial-ulnar axes had significantly lower PSDs, between 10.9 and 25.2°. The ulnar-radial axes were lower in PSD value but also in uncertainty window range. This addresses the concern over the asymmetry of PSDs in Table 1, as the narrow range supports better detection even if it is slightly offset. Results indicate that increased detection ability occurs in the radial-ulnar axis, referenced to the orientation of the hand rather than the environment. To arrive at these conclusions, we find it important to address the statistical limitations of our analysis and process. While analyzing primary stimuli directions independently, limited or no significant results were found. However, grouping the primary stimuli into primary axes provides significant anisotropic differences. While this is likely exacerbated by the limited sample size, we believe this provides insight toward subjective understanding of somatosensory and proprioceptive integration.

From the analysis, heightened directional discrimination ability in the radial-ulnar axis supports that this discrimination is referenced to the subjects’ hand orientation, and we offer the explanation of increased distal-phalange movement in that axis. Statistically, the DP1 and DP2 demonstrated increased movement during trials in the radial-ulnar axis. Therefore, the increased detection ability could be explained by these movements, even when the significant differences are less than a millimeter. While the gripped cube moved 10 mm and the motion capture only indicates DP movement less than 3 mm, it is clear that proprioceptive information from these significant displacements is the primary explanation for increased detection ability.

As a consideration, the results are likely not solely due to displacement as the majority of object movement, near 70%, is left to be absorbed and interpreted with other means. Multisensory integration of somatosensation and proprioception is occurs even at miniscule additions of tactile information, so it is not unreasonable to think the combination of miniscule but significant joint displacement and complex tactile information are both being utilized (Rincon-Gonzalez et al., 2011). The somatosensation component could be explained by surface friction inducing skin deformation. Passive slip literature indicates that slip texture, speed, and direction sensitivity should exist in the distal-proximal axis, potentially attributed to factors such as the anisotropic properties of the fingertip surface’s dermal ridging (Maeno and Kobayashi, 1998; Maeno et al., 1998). However, the finger pad is also anisotropic in its ability to deform, and the combination of dermal ridging and glabrous tissue interactions may provide an answer. Somatosensory cortex possesses multimodal representations of passive lateral finger displacement and cutaneous touch (Kim et al., 2015). Contextual activation of the finger pad informs these multimodal representations during precision grip cued by weight, texture, and increased friction (Salimi et al., 1999a,b,c). The different cues are directionally influenced by the anisotropic properties of the finger pads’ glabrous skin. First, papillary ridges at the middle of the finger are orthogonal to the radial-ulnar axis, predicting increased deformation in the respective axis. With increased skin stretch comes increased perception of tactile information (Wang and Hayward, 2007; Provancher and Sylvester, 2009; Seizova-Cajic et al., 2014). During the previously observed passive slip tasks, the glabrous skin is not likely heavily engaged and the tactile stimuli are superficial. By utilizing precision grip, our task engages more of the inherent biomechanical properties of the fingers and fingertips. Directional grip detection sensitivity, but not superficial slip sensitivity, is a function of the amount of observed digit displacement and, potentially, skin stretch in respective directions. Further investigation focused on the latter variable.

## Conclusion

Precision grip responses are modulated by task context as seen in forces, latencies, and orientation sensitivity. We studied the perception of the task not the response properties, and observed an internally referenced source of information independent of hand posture. This tactile directional discrimination is claimed to be biomechanically referenced as a result of increased digit movement and hypothesized to be influenced by anisotropic fingerpad properties. These are not inherently competing results but argue that perceptual responses stem from directionally dependent activation of the parallel inputs.

## Data Availability Statement

The raw data supporting the conclusions of this article will be made available by the authors, without undue reservation.

## Ethics Statement

The studies involving human participants were reviewed and approved by the Arizona State University Institutional Review Board. The patients/participants provided their written informed consent to participate in this study.

## Author Contributions

JT and NN contributed to the experimental design, data collection, analysis of the data, and preparation of the manuscript. SH provided mentorship and advice, as well as editing and oversight. All authors contributed to the article and approved the submitted version.

## Conflict of Interest

The authors declare that the research was conducted in the absence of any commercial or financial relationships that could be construed as a potential conflict of interest.
